# Comprehensive Multi‐Omics Analysis of Copper Metabolism Related Molecular Subtypes and Prognostic Risk Stratification in Colon Adenocarcinoma

**DOI:** 10.1111/jcmm.70591

**Published:** 2025-05-20

**Authors:** Xi Sun, Jingfei Tong, Xiaojie Fang, Miaojiong Lu, Chunhui Rao, Yanyan Li

**Affiliations:** ^1^ Department of Anorectal Surgery Hangzhou TCM Hospital Affiliated to Zhejiang Chinese Medical University Hangzhou China

**Keywords:** colon adenocarcinoma, copper metabolism‐related genes, COX19, risk stratification, single‐cell sequencing

## Abstract

Colon adenocarcinoma (COAD) is the most common subtype of colorectal cancer, originating from glandular cells in the colon. Despite diagnostic and therapeutic advances, its prognosis remains poor. Copper, an essential micronutrient, is involved in tumorigenesis and other biological processes. In this study, we identified copper metabolism‐related genes (CMRG) associated with COAD prognosis from TCGA and GEO databases and constructed a CMRG‐based risk model. We assessed its clinical relevance through analyses of immune infiltration, immunotherapy response, and drug sensitivity. Single‐cell sequencing revealed the spatial and cellular distribution of CMRG in COAD tissues, providing insight into their roles in the tumour microenvironment. COX19 was selected for further validation, and in vitro experiments (western blot, PCR, siRNA, colony formation, and Transwell assays) confirmed its role in promoting COAD cell invasion and proliferation. These findings highlight the involvement of copper metabolism in COAD progression and suggest potential targets for therapy.

**Abbreviations**
AOC2: Amine Oxidase Copper Containing 2
APC: Adenomatous Polyposis Coli
ATCC: American Type Culture Collection
ATP7A: ATPase Copper Transporting Alpha Polypeptide
CCK‐8: Cell Counting Kit‐8
CMRG: Copper Metabolism‐Related Genes
COAD: Colon Adenocarcinoma
COX19: Cytochrome c Oxidase Assembly Factor 19
CTLA4: Cytotoxic T‐Lymphocyte‐Associated Protein 4
EMT: Epithelial‐Mesenchymal Transition
ESTIMATE: Estimation of Stromal and Immune Cells in Malignant Tumour Tissues
FDR: False Discovery Rate
FPKM: Fragments Per Kilobase of Transcript per Million Mapped Reads
GAPDH: Glyceraldehyde‐3‐Phosphate Dehydrogenase
GEO: Gene Expression Omnibus
GO: Gene Ontology
GSVA: Gene Set Variation Analysis
HR: Hazard Ratio
IC50: Half‐Maximal Inhibitory Concentration
IPS: Immunophenoscore
KEGG: Kyoto Encyclopedia of Genes and Genomes
LASSO: Least Absolute Shrinkage and Selection Operator
MSI‐H: Microsatellite Instability‐High
MSS: Microsatellite Stable
PCA: Principal component analysis
PCA: Principal Component Analysis
ROS: Reactive Oxygen Species
SDS‐PAGE: Sodium Dodecyl Sulfate‐Polyacrylamide Gel Electrophoresis
ssGSEA: Single‐Sample Gene Set Enrichment Analysis
TPM: Transcripts Per Million
t‐SNE: t‐Distributed Stochastic Neighbour Embedding
UMAP: Uniform Manifold Approximation and Projection

## 1 Introduction

Colon adenocarcinoma (COAD) is a malignant tumour originating from the glandular cells of the colon's inner lining and is the most common type of colorectal cancer [1]. Clinically, COAD has a high incidence and mortality, particularly in developed countries [2]. Despite advancements in diagnostic and therapeutic approaches, the prognosis for COAD remains unsatisfactory [3, 4]. Further mechanistic studies are essential to elucidate the molecular basis of its occurrence and progression, which can lead to the development of more effective targeted therapies [5]. Additionally, risk stratification enables more accurate prediction of patient outcomes, optimising individualised treatment plans and improving patient survival rates and quality of life [6].

As an important cofactor of mammalian enzymes, copper occurs in two oxidation states (Cu^1+^ and Cu^2+^) and is primarily distributed in the muscles, liver, and brain [7]. As an essential micronutrient, copper is involved in a variety of biological processes including mitochondrial respiration, antioxidant defence, and bio‐compound synthesis [8]. Copper homeostasis is regulated by multiple key molecules, including many key molecular targets, such as ceruloplasmin, CTR1, SLC31A1, ATOX1 [9, 10]. Dysregulation of copper homeostasis can lead to oxidative stress and cytotoxicity [8, 11, 12]. Copper metabolism has been implicated in multiple physiological and pathological processes, including aging, oxidative stress, inflammation, and immune dysregulation [13, 14].

Copper metabolism is believed to be involved in carcinogenesis due to its influence on signalling pathways [15]. It plays a vital role in tumour development, primarily by promoting angiogenesis [16]. In addition, copper affects the spread and formation of secondary tumours by activating enzymes responsible for cell proliferation [17]. Copper imbalance alters metabolic reprogramming in tumour cells by affecting glycolysis, insulin resistance, and lipid metabolism [18, 19]. Copper plays a key role in receptor tyrosine kinase signalling, thereby promoting malignant cell growth and proliferation [20]. Various carcinogenic signalling pathways such as PI3K‐AKT, ULK1/2, BRAF, NF‐kB are influenced by copper and further affect tumour progression [21, 22, 23, 24]. Preliminary evidence also suggests the potential role of copper metabolism in colon cancer. Multiple regional reports suggest that increased levels of copper and ceruloplasmin are significantly associated with the risk of colon cancer [25, 26, 27]. Therefore, risk stratification of COAD based on copper metabolism related targets has positive academic value and potential clinical significance.

In this study, we screened copper metabolism‐related genes (CMRG) associated with COAD prognosis from TCGA and GEO public databases and performed molecular typing. The subsequent establishment of the CMRG scoring system model confirmed the possibility of prognostic stratification of COAD by CMRG. The analysis of tumour immune microenvironment (TME) infiltration and immunotherapy response highlights the clinical significance of the CMRG score model. Analysis of single‐cell sequencing data enhances our understanding of the role of CMRG in different cellular environments and the potential impact of targeted therapies. In vitro experiments such as western blot, PCR, siRNA, cell cloning, and Transwell assay verified the role of the screened target cytochrome c oxidase 19 (COX19) in COAD, thus partially confirming the reliability of the bioinformatics analysis results. Our results provide a new basis for the involvement of copper metabolism in the pathogenesis of COAD.

## 2 Materials and Methods

### 2.1 Acquisition and Standardised Processing of Microarray Data

Based on the independently available TCGA and GEO databases, we downloaded and collected transcriptomic microarray data and clinical baseline characteristics of COAD samples. In the Perl programming environment, we preprocessed the transcriptomic matrices of normal and COAD samples from the TCGA database and performed annotation analysis based on gene annotation files. From the GEO database, we downloaded the external cohort dataset GSE39582 and annotated gene tags using the corresponding platform annotation file (GPL570, [HG‐U133 Plus 2] Affymetrix Human Genome U133 Plus 2.0 Array). According to the clinical baseline characteristics provided by the two independent databases, after excluding samples with missing survival information, we extracted 41 normal samples and 446 COAD samples from the TCGA database for subsequent analysis. Additionally, we extracted 562 COAD samples from the GSE39582 dataset for further analysis. To eliminate batch effects between the transcriptomic matrices from the two different databases, we first used the ‘limma’ script to convert the transcriptomic data of COAD samples from the TCGA database from FPKM format to TPM format. Subsequently, we applied the ‘sva’ R package to standardise the transcriptomic data and correct for batch effects between the TCGA and GEO databases and used the ‘ggplot2’ R package to generate principal component analysis (PCA) plots before and after batch effect correction (Figure S1A,B).

### 2.2 Differential Expression Analysis of CMRG in COAD

Based on previously reported literature, we collected 133 CMRG for subsequent investigation (Table S1). Using a differential threshold of |fold change| > 1 and *p*.adjust < 0.05, we analysed the differential expression of the CMRG signature between normal and COAD samples using the ‘limma’ script. The ‘pHeatmap’ script was utilised to visualise the expression of DE‐CMRG between normal and COAD samples. Additionally, we employed the ‘clusterProfiler’ R script to perform GO and KEGG enrichment analyses of the DE‐CMRG signature, aiming to explore potential molecular regulatory mechanisms.

### 2.3 Identification of Prognosis‐Related CMRG Signatures and Molecular Subtype Characterisation

Based on survival data from COAD, we performed univariate Cox analysis to evaluate the prognostic value of DE‐CMRG signatures in COAD. Using the ‘glmnet’ R script, we constructed a LASSO function model to select prognostic CMRG variables for further analysis. Through multivariate Cox analysis, we further assessed the independent prognostic value of these CMRG variables and determined the molecular subtype characteristics of COAD samples based on the expression levels of these independent prognostic variables. Using the ‘ConsensusClusterPlus’ R script, we applied the k‐means algorithm to divide COAD samples into clusters with classification ratios (K) ranging from 2 to 9 and calculated the model parameters for each k value. Based on the optimal k and model parameters, we identified CMRG molecular subgroups in COAD samples and visualised them using a PCA plot generated with the ‘ggplot2’ package to evaluate the distinctiveness of the CMRG subtypes. To explore differential regulatory KEGG signalling pathways between CMRG subgroups, we employed the ‘GSVA’ algorithm using the KEGG reference gene set ‘c2.cp.kegg.v7.2.symbols.gmt.’

### 2.4 Construction and Validation of the CMRG Scoring System Model

Based on the expression levels of independent prognostic CMRG signatures and the risk values calculated from multivariate Cox analysis, we calculated the CMRG scores for each COAD sample and constructed the CMRG scoring system. The CMRG score formula is as follows: CMRG Score = Expression Level of CMRG Prognostic Variables × Risk coefficient. Using the ‘caret’ algorithm, we divided the COAD samples into training and validation sets in a 6:4 ratio and calculated the CMRG scores for each independent cohort. COAD samples in each cohort were stratified by risk according to the median CMRG score, and survival curves were plotted using the ‘survival’ R script to assess the stability and accuracy of the CMRG scoring system in predicting COAD clinical outcomes. Additionally, we used the GSE39582 dataset as an external validation cohort, calculated the CMRG scores for the samples, and analysed the clinical survival outcomes of the CMRG score subgroups to validate the independence of the CMRG scoring system. Finally, we used the ‘ggalluvial’ R script to create Sankey diagrams to explore the associations between CMRG molecular subtypes, the CMRG scoring system, and COAD clinical prognosis. Based on the ‘survival’ R package, we performed univariate and multivariate Cox regression analyses to evaluate the independent prognostic value of each clinicopathological variable and the CMRG score in two independent datasets (TCGA and GSE39582).

### 2.5 Immune Microenvironment Infiltration Characteristics and Evaluation of Immunotherapy Response

Based on the transcriptomic characteristics of COAD samples, we assessed the immune infiltration status using the ‘estimate’ script and quantitatively calculated four immune infiltration indicators: stromal score, immune score, ESTIMATE score, and tumour purity. Using marker genes for 23 immune cell types, we performed ssGSEA enrichment analysis with the ‘GSVA’ algorithm to score the relative proportions of these 23 immune infiltrating cells. The ‘limma’ script was used for statistical analysis of differences in immune infiltration proportions among different subgroups, and the ‘ggplot2’ script was used to calculate the correlation (Pearson correlation) between CMRG prognostic factors and immune infiltration. Using the IMvigor210 database, we evaluated the response of COAD samples from different subgroups to PD‐L1 immunotherapy. Additionally, based on the TCIA database, we analysed the response outcomes to PD1/CTLA4 immunotherapy in different subgroups. In the Perl programming environment, we extracted somatic mutation data (in maf format) of COAD samples from the TCGA database and used the ‘maftools’ R script to plot the somatic mutation landscape for CMRG score subgroups.

### 2.6 Single‐Cell Sequencing Data Analysis

The single‐cell RNA sequencing data used in this study were obtained from the GEO database. After excluding other samples, we retrieved single‐cell sequencing data (10× Genomics) from 3 normal samples and 6 COAD tissue samples in the GSE231559 dataset for subsequent analysis. Using the ‘Seurat’ R package, we performed quality control on each sample, excluding low‐quality cells characterised by UMI counts below 500 or a mitochondrial gene expression ratio exceeding 10%. The LogNormalize method was applied to normalise gene expression data for each cell, and the FindVariableFeatures function was used to identify highly variable genes. Batch effect correction was conducted using the Mutual Nearest Neighbours (MNN) method within the Seurat package. Subsequently, PCA was employed for initial dimensionality reduction, and the top 20 principal components were selected for downstream analysis. Based on the PCA results, we performed two‐dimensional visualisation using UMAP and t‐SNE methods. Cell clustering analysis was conducted using the Louvain algorithm based on nearest neighbours, with a resolution parameter set to 0.8. The FindClusters function was used to identify cell subpopulations, and cell types were annotated using known marker genes and the SingleR public database. Visualisation was carried out using the “ggplot2” and ‘Seurat’ packages, generating UMAP/t‐SNE plots, heatmaps, and violin plots to present key findings.

### 2.7 Cell Culture

The human colon cancer cell lines HCT116 and SW480, as well as the human normal colonic mucosal epithelial cell line NCM460, were obtained from the American Type Culture Collection (ATCC). HCT116 cells were cultured in RPMI 1640 medium (Gibco, USA) supplemented with 10% fetal bovine serum (FBS, Gibco, USA) and 1% penicillin–streptomycin (P/S, Gibco, USA). SW480 cells were maintained in Leibovitz's L‐15 medium (Gibco, USA) supplemented with 10% FBS and 1% P/S (Gibco, USA). NCM460 cells were cultured in DMEM medium (Gibco, USA) with 10% FBS and 1% P/S (Gibco, USA). HCT116 and NCM460 cells were incubated at 37°C with 5% CO_2_, whereas SW480 cells were maintained at 37°C in an atmosphere without CO_2_, as recommended for L‐15 medium. The culture medium was refreshed every 2–3 days. When the cells reached 80%–90% confluence, they were digested with 0.25% trypsin (Gibco, USA) and passaged for further experiments.

### 2.8 Western Blot Analysis

Total protein was extracted from NCM460, HCT116, and SW480 cells using RIPA lysis buffer (Beyotime, China) supplemented with protease inhibitors (Roche, Switzerland). Protein concentration was determined using the BCA method (Beyotime, China). Equal amounts of protein samples (20–30 μg) were separated on a 10% SDS‐PAGE gel and then transferred onto PVDF membranes (Millipore, USA). The membranes were blocked at room temperature for 1 h in TBST solution containing 5% non‐fat milk. Subsequently, the membranes were incubated overnight at 4°C with a COX19 antibody (Invitrogen, 1:1000). The following day, the membranes were incubated at room temperature for 1 h with an HRP‐conjugated secondary antibody (Abcam, 1:5000). The bands were visualised using ECL chemiluminescent reagents (Millipore, USA) and imaged. The band intensity was analysed using ImageJ software, and the expression levels were normalised to the internal control protein (GAPDH, Abcam).

### 2.9 Real‐Time Quantitative PCR (RT‐qPCR) Analysis

Total RNA was extracted from NCM460, HCT116, and SW480 cells using Trizol reagent (Invitrogen, USA). The concentration and purity of the extracted RNA were measured using a NanoDrop 2000 spectrophotometer (Thermo Fisher Scientific, USA), ensuring an A260/A280 ratio between 1.8 and 2.0. A total of 1 μg of RNA was reverse transcribed into cDNA using the PrimeScript RT Reagent Kit (TaKaRa, Japan), following the manufacturer's instructions. The qPCR detection of the COX19 gene was performed using the SYBR Premix Ex Taq II Kit (TaKaRa, Japan). The primers were synthesised by Sangon Biotech (Shanghai, China) with the following sequences: COX19 Forward Primer: 5′‐CCATTTGCAGTTAGGCTCGC‐3′; COX19 Reverse Primer: 5′‐CAGGCAACTTTGGCACAGAC‐3′. The relative expression of the COX19 gene was calculated using the $2^{-\Delta \Delta C_{t}}$ method, with GAPDH serving as the internal reference gene for normalisation.

### 2.10 CCK‐8 Assay

NCM460, HCT116, and SW480 cells were seeded at a density of 5000 cells per well in a 96‐well plate, with three replicates per group. The cells were incubated at 37°C with 5% CO_2_ for 24 h to allow adhesion. Following the instructions of the CCK‐8 assay kit (Dojindo, Japan), 10 μL of CCK‐8 solution was added to each well. After gentle mixing, the plate was incubated in the incubator for an additional 2 h. Absorbance values (OD values) at a wavelength of 450 nm were measured using a microplate reader (Multiskan FC, Thermo Fisher Scientific, USA). The proliferation rate of each cell group was calculated based on the OD values, and the relative proliferation rate of the treatment groups was compared to the control group.

### 2.11 Construction and Transfection of COX19 Interference Plasmid in HCT116 Cells

The designed siRNA sequences were cloned into the pSilencer 2.1‐U6 hygro plasmid vector (Invitrogen, USA). Restriction endonucleases and DNA ligase were used to insert the siRNA sequences into the vector, creating a plasmid containing the COX19 interference sequence. The constructed COX19 interference plasmid was then transfected into HCT116 and SW480 cells. One day before transfection, HCT116 and SW480 cells were seeded in a 6‐well plate to ensure that the cells reached 70%–80% confluence at the time of transfection. Transfection was performed using Lipofectamine 3000 transfection reagent (Thermo Fisher Scientific, USA). According to the manufacturer's instructions, 1 μg of interference plasmid DNA was mixed with 3 μL of Lipofectamine 3000 reagent, and the mixture was incubated at room temperature for 20 min before being added to the cell culture medium of HCT116 and SW480 cells. The cells were then incubated in the incubator for 24 h. After 24 h, the original medium was replaced with fresh medium, and the cells continued to be cultured at 37°C with 5% CO_2_.

### 2.12 Cell Colony and Transwell Assay

HCT116 and SW480 cells and siCOX19‐interfered HCT116 and SW480 cells were seeded at a density of 500 cells per well in a 6‐well plate and cultured until they adhered and grew to approximately 70%–80% confluence. The cells were incubated in a 37°C, 5% CO_2_ incubator for 1–2 weeks until visible colonies formed. After the incubation period, the cells were fixed with 4% paraformaldehyde (Sigma‐Aldrich, USA) for 15 min and then stained with a 0.1% crystal violet (Crystal Violet, Sigma‐Aldrich, USA) solution for 10 min. Unbound dye was washed away with PBS. Colonies were observed under a microscope (Leica, Germany), and the number of colonies was counted to assess cell proliferation ability. For the Transwell assay, HCT116, SW480 cells, and siCOX19‐interfered HCT116, SW480 cells were cultured in appropriate medium until they reached the logarithmic growth phase. Cells were trypsinized (Trypsin, Gibco, USA), washed, and resuspended in serum‐free medium to adjust to the desired cell density (1 × 10^5^ cells/mL). Transwell chambers (Corning, USA) with an 8 μm pore size filter were used. Cell suspensions (100 μL, 1 × 10^5^ cells) were added to the upper chamber, while the lower chamber contained medium with 10% fetal bovine serum (FBS, Gibco, USA) as a chemoattractant. The chambers were placed in a 37°C, 5% CO_2_ incubator for 24 h. After migration, the Transwell chambers were removed, and non‐migrated cells were washed away with PBS. Cells were fixed with 4% paraformaldehyde for 15 min and stained with a 0.1% crystal violet solution for 10 min. Unbound dye was washed away with PBS. Cells that migrated to the underside of the membrane were observed and counted under a microscope (Leica, Germany) to evaluate cell migration ability. Images can be captured under the microscope and quantitatively analysed if needed.

### 2.13 Statistical Analysis

In this study, we utilised R and Perl for preprocessing the raw data, which included data cleaning, handling missing values, and standardisation. Statistical comparisons between two groups were performed using the Wilcoxon rank‐sum test to assess the significance of group differences. For comparisons among multiple groups, One‐way ANOVA was employed to evaluate differences between groups. Cell experiment data were analysed from three independent replicates. All statistical results were adjusted for multiple comparisons to control the false discovery rate (FDR). The Benjamini‐Hochberg method was used for FDR control to ensure the reliability of the results. The significance level for all statistical tests was defined as *p* < 0.05. Data are presented as mean ± standard deviation (SD). Significance is indicated as follows: *p < 0.05; **p < 0.01; ***p < 0.001.

## 3 Results

### 3.1 Differential Expression Analysis and Molecular Mechanism of CMRG Signatures in COAD

**FIGURE 1:**
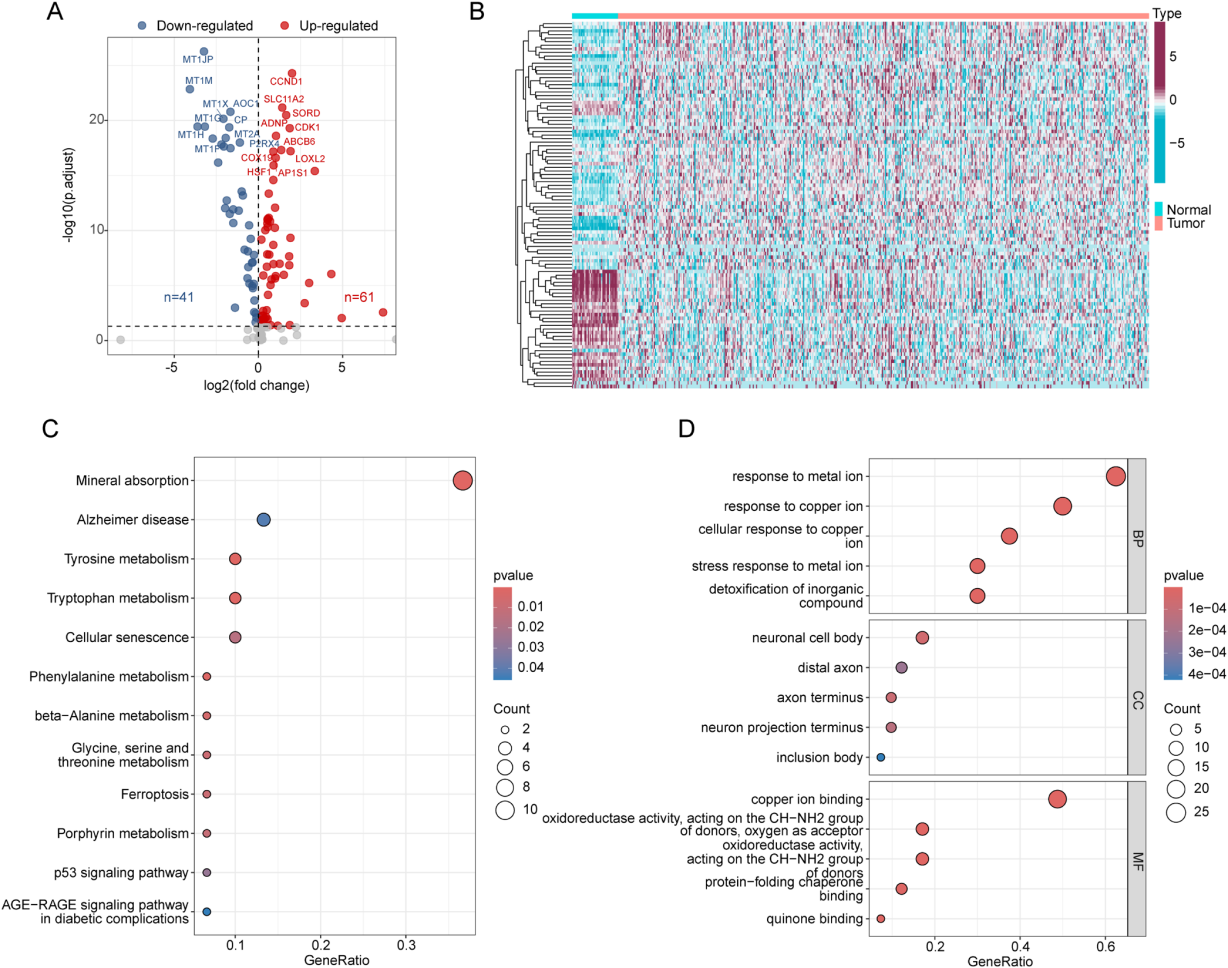
Differential expression identification and potential molecular function prediction of CMRG signatures in COAD. (A) Differential expression analysis of CMRG signatures between normal and COAD samples. Differential thresholds: |FC| > 1 and *p*.adjust < 0.05. Red points indicate upregulation, while blue points indicate downregulation. (B) Heatmap visualisation analysis of differentially expressed CMRG between normal and COAD samples. (C, D) Molecular function mechanism analysis of differentially expressed CMRG.

In this study, a total of 133 CMRG signatures were included to elucidate potential regulatory roles in COAD (Figure S2). Differential expression analysis revealed that 41 CMRG signatures were significantly downregulated, while 61 CMRG signatures were significantly upregulated in COAD (Figure 1A,B). To preliminarily assess the potential molecular mechanisms of these differentially expressed CMRG signatures, we performed KEGG and GO analyses. As shown in Figure 1C,D, KEGG analysis revealed that these DE‐CMRG signatures are associated with pathways such as mineral absorption, Alzheimer's disease, tyrosine metabolism, tryptophan metabolism, and cellular senescence. GO analysis suggested that DE‐CMRG signatures may be associated with functions related to response to metal ions, response to copper ions, cellular response to copper ions, neuronal cell body, and copper ion binding.

### 3.2 Identification of Prognostic CMRG Signatures and Molecular Subtype Characterisation

**FIGURE 2:**
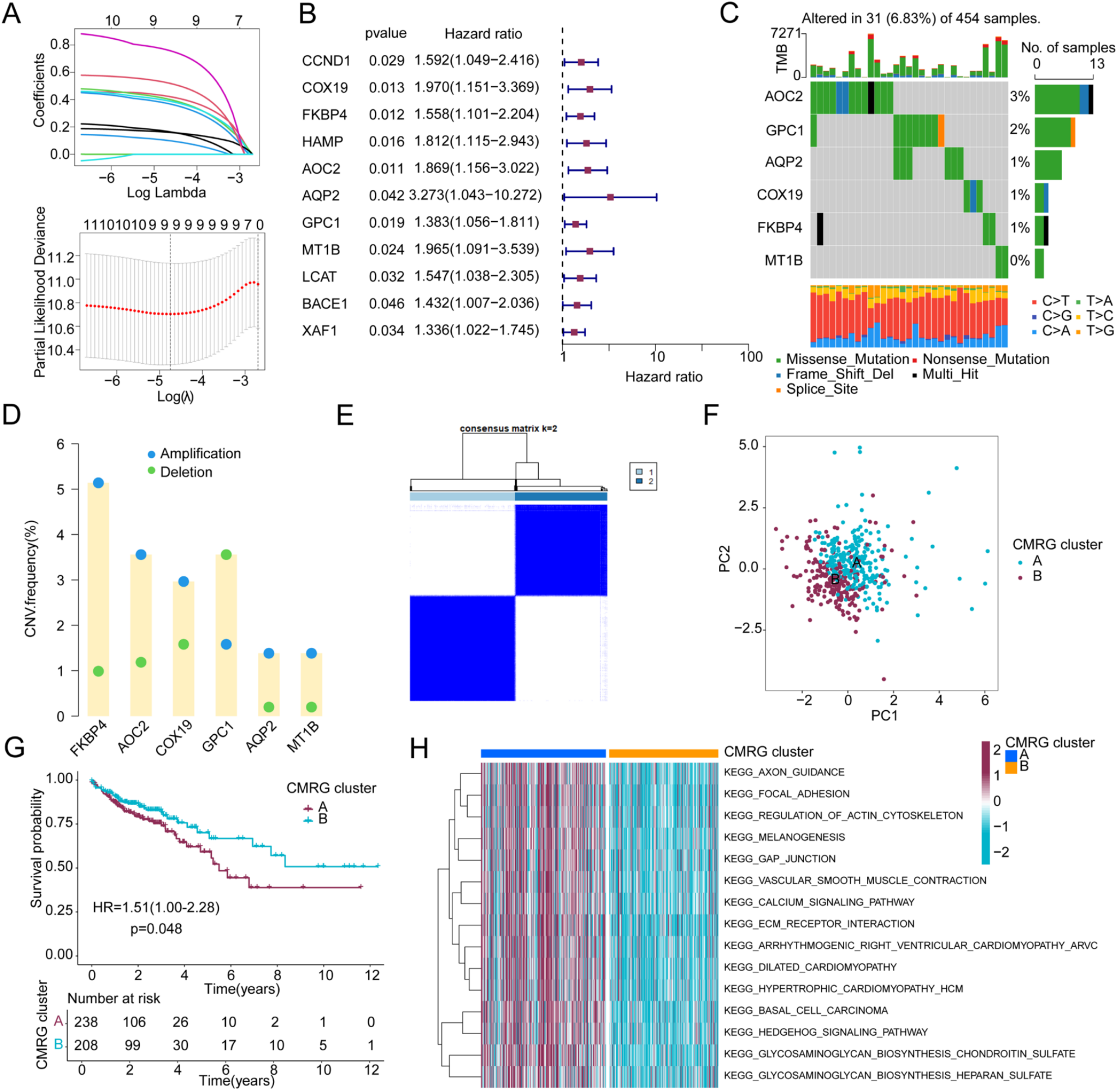
Identification of prognostic‐related CMRG signatures and molecular subtype feature analysis. (A, B) Identification of prognostic‐related CMRG signatures in COAD using the LASSO‐univariate Cox analysis algorithm. (C) Analysis of somatic mutation frequency in CMRG prognostic signatures. (D) Analysis of copy number variation frequency in prognostic‐related CMRG. (E) Molecular subtype identification based on prognostic‐related CMRG signatures. (F) PCA plot showing the distribution characteristics of CMRG molecular subgroups. (G) KM curve analysis of CMRG molecular subtypes. (H) Differential analysis of KEGG signalling pathways in CMRG molecular subgroups.

To elucidate the potential association between CMRG signatures and clinical prognosis in COAD, we calculated the hazard ratios (HR) and *p*‐values for each CMRG using univariate Cox analysis. The results indicated that 11 CMRG signatures were associated with poor prognosis in COAD. Using the LASSO algorithm, we further identified 9 prognostic CMRG signatures for subsequent analysis (Figure 2A,B). Multivariate Cox analysis was then performed to evaluate the independent prognostic value of these 9 signatures. The analysis identified six CMRG signatures (AOC2, GPC1, AQP2, COX19, FKBP4, and MT1B) as independent prognostic factors for COAD. Mutation frequency analysis showed that AOC2 had a mutation rate of 3%, followed by GPC1 (2%), AQP2 (1%), COX19 (1%), and FKBP4 (1%) in COAD (Figure 2C). Copy number variation analysis indicated that AOC2, AQP2, COX19, FKBP4, and MT1B exhibited significant copy number amplifications, while GPC1 showed notable copy number deletions in COAD (Figure 2D). To better understand the role of these prognostic CMRG signatures in COAD, we performed consensus clustering based on the expression profiles of the 6 independent prognostic CMRG signatures. Using the optimal model parameter (k), we classified COAD samples into two distinct CMRG molecular subtypes: CMRG subtype A (238 samples) and CMRG subtype B (208 samples) (Figure 2E). PCA plots demonstrated a clear separation between the two CMRG subtypes, indicating significant differences between COAD samples in each subtype (Figure 2F). Clinical outcome analysis indicated that COAD samples in CMRG subtype B had significantly better survival outcomes than those in subtype A (Figure 2G, *p* = 0.048, HR = 1.51(1.00–2.28)). GSVA analysis provided preliminary insights into the potential regulatory mechanisms between the CMRG molecular subtypes. KEGG results revealed that pathways such as focal adhesion, regulation of actin cytoskeleton, melanogenesis, and calcium signalling were significantly upregulated in the clinically poorer CMRG subtype A, potentially serving as key signalling pathways regulating the clinical survival differences between the CMRG subtypes (Figure 2H).

### 3.3 Immune Microenvironment Infiltration Characteristics and Immunotherapy Response Prediction Analysis of CMRG Molecular Subtypes in COAD

**FIGURE 3:**
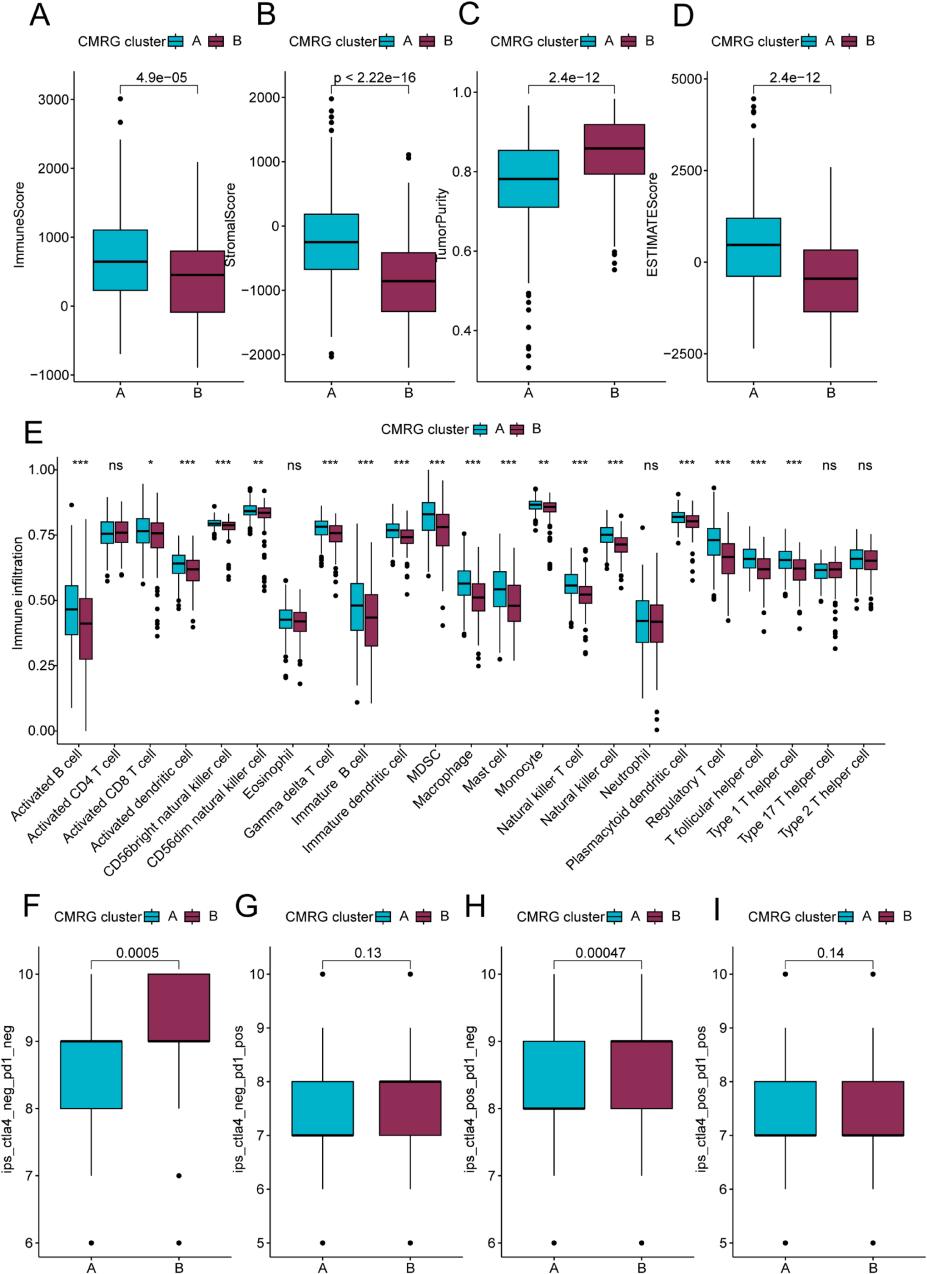
Immune microenvironment infiltration characteristics and immune therapy response analysis of CMRG molecular subgroups. (A–D) Assessment of immune infiltration status based on the ESTIMATE algorithm. (E) Differential analysis of the proportion of 23 immune cell types in CMRG molecular subgroups using the ssGSEA algorithm. (F–I) IPS scores revealing the response of CMRG scoring subgroups to PD‐1/CTLA4 immune therapy.

In the subsequent analysis, we further evaluated the immune microenvironment infiltration characteristics of different CMRG molecular subtypes in COAD. CMRG subtype A exhibited significantly higher immune scores, stromal scores, and ESTIMATE scores compared to CMRG subtype B, while tumour purity was notably lower (Figure 3A–D). These findings suggest that there may be significant differences in the immune microenvironment between CMRG molecular subtypes. Next, we used the ssGSEA algorithm to quantitatively assess the infiltration proportions of 23 immune cell types between the CMRG subtypes. The results indicated that CMRG subtype A had a significantly higher infiltration proportion of immune cells, including activated B cells, activated dendritic cells, immature B cells, macrophages, and MDSCs, compared to CMRG subtype B (Figure 3E). Furthermore, we evaluated the response of CMRG molecular subtypes to PD1 and CTLA4 immunotherapy using the TCIA database. Immunophenoscore (IPS) results indicated that CMRG subtype B might show a greater response to PD1 and CTLA4 immunotherapy compared to CMRG subtype A (Figure 3F–I).

### 3.4 Development and Validation of CMRG Related Prognostic Scoring System in COAD

**FIGURE 4:**
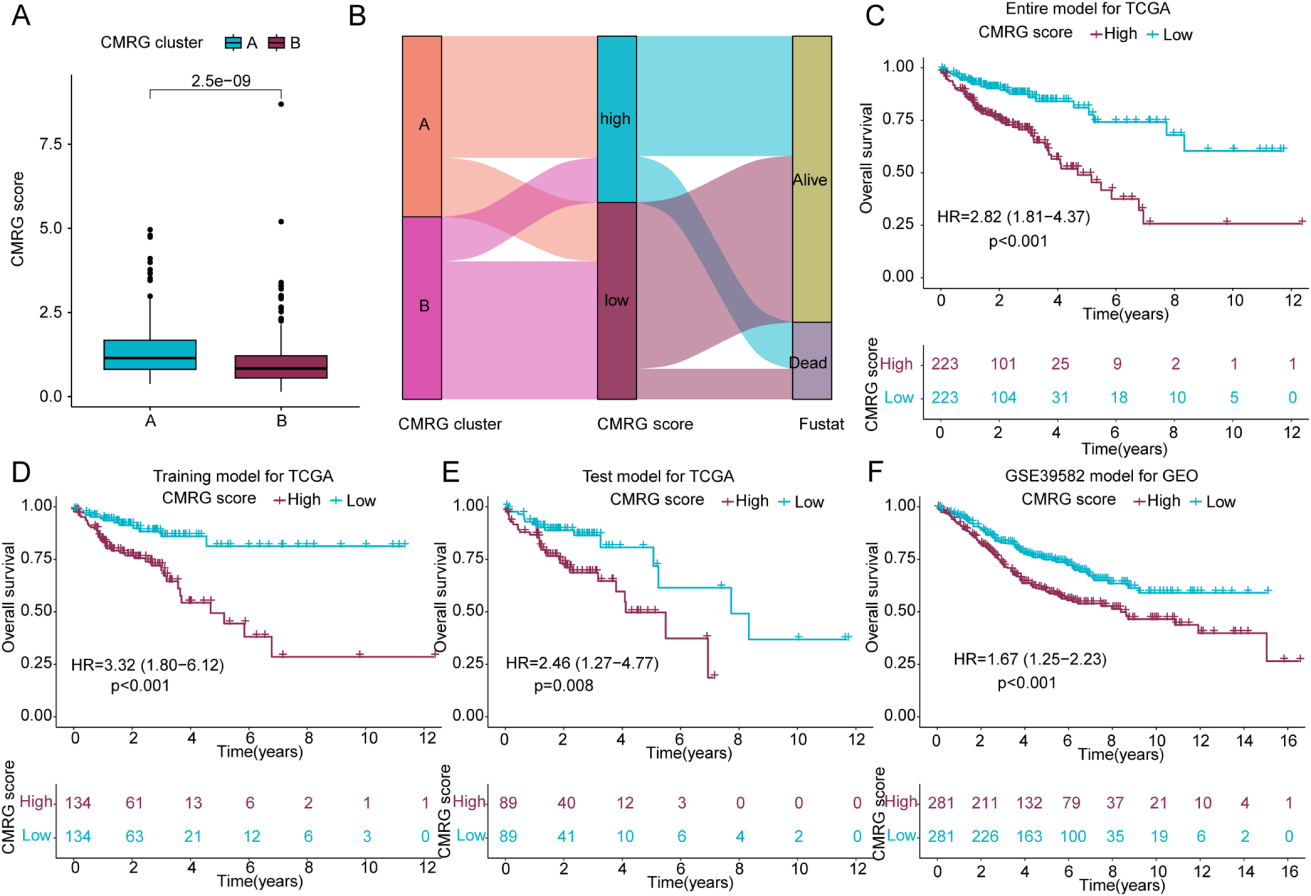
Construction and independent validation of the CMRG scoring system. (A) Differential analysis of CMRG scores among CMRG molecular subgroups. (B) Sankey diagram illustrating the potential connections between CMRG molecular subgroups, CMRG scoring system, and clinical prognosis in COAD. (C‐E) Clinical prognosis analysis of CMRG scoring subgroups in the complete cohort, training cohort, and validation cohort of the TCGA dataset. (F) Clinical survival prognosis analysis of CMRG scoring subgroups in the external cohort GSE39582 dataset.

A CMRG scoring system was developed based on the independent prognostic CMRG signatures. The differential analysis results indicated that CMRG scores were significantly higher in the poorer prognosis CMRG subtype A compared to subtype B, suggesting that higher CMRG scores may be associated with worse prognosis in COAD (Figure 4A). Using a Sankey diagram, we illustrated the potential connections between CMRG molecular subtypes, the CMRG scoring system, and COAD clinical prognosis. The results showed that the better prognosis CMRG subtype B was associated with lower CMRG scores, while the poorer prognosis CMRG subtype A was associated with higher CMRG scores (Figure 4B). To validate the accuracy and reliability of the CMRG scoring system in predicting COAD clinical outcomes, we randomly divided COAD samples into training and validation sets in a 6:4 ratio and categorised each cohort into CMRG scoring subgroups based on the median CMRG score. In the complete TCGA dataset, we observed that COAD samples with low CMRG scores had significantly better clinical outcomes compared to those with high CMRG scores (Figure 4C, *p* < 0.001, HR = 2.82(1.81–4.37)). This trend was also observed in both the training and validation sets of the TCGA dataset, where low CMRG scoring subgroup COAD samples showed markedly better clinical outcomes than high CMRG scoring subgroup samples (Figure 4D,E, *p* < 0.001, HR = 3.32(1.80–6.12); *p* = 0.008, HR = 2.46(1.27–4.77)). Additionally, in an external independent cohort GSE39582, we further validated the stability of the CMRG scoring system in predicting COAD clinical outcomes. Survival curve results indicated that the low CMRG scoring subgroup had significantly better clinical survival outcomes compared to the high CMRG scoring subgroup (Figure 4F, *p* < 0.001, HR = 1.67(1.25–2.23)). In the TCGA dataset, univariate Cox analysis indicated that stage, T stage, and the CMRG score were associated with poor prognosis in COAD (Figure S3A). Multivariate Cox analysis further revealed that the CMRG score might serve as an independent prognostic factor for COAD (Figure S3B). Notably, in the independent external cohort GSE39582, we also found that the CMRG score was independently associated with poor prognosis in COAD (Figure S3C,D). These results conclude that the CMRG scoring system can effectively stratify COAD risk and accurately assess clinical survival outcomes in COAD. To explore the translational potential of the CMRG score, we next analysed its correlation with immune microenvironment features and drug sensitivity.

### 3.5 Immune Microenvironment Characteristics and Drug Sensitivity Analysis of CMRG Scoring Subgroups in COAD

**FIGURE 5:**
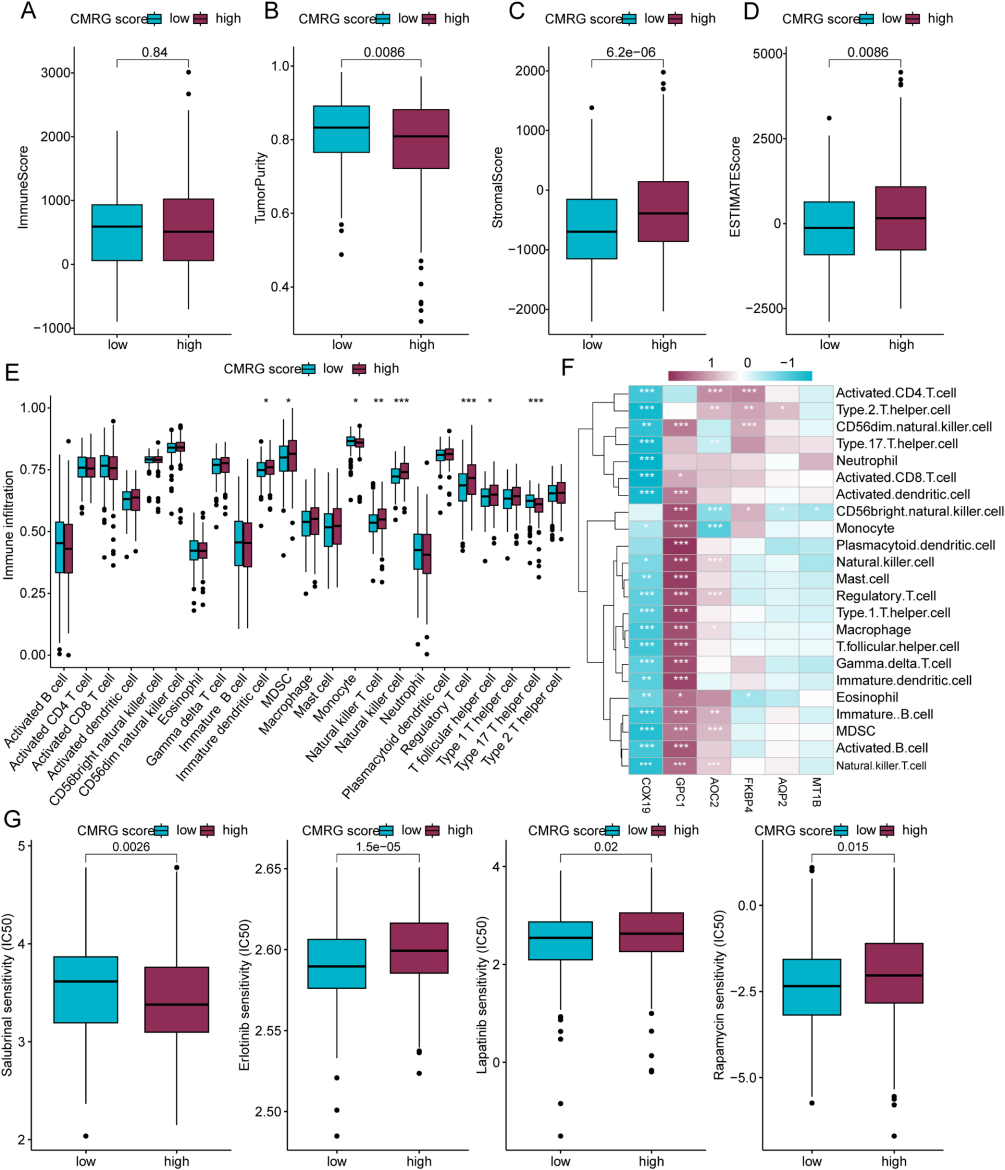
Immune microenvironment infiltration characteristics and drug sensitivity prediction analysis of CMRG scoring subgroups. (A‐D) Assessment of immune infiltration status in CMRG scoring subgroups. (E) Differential analysis of the proportion of 23 immune cell types in CMRG scoring subgroups. (F) Correlation analysis between CMRG prognostic signatures and 23 immune cell types. (G) Potential drug sensitivity analysis of CMRG scoring subgroups.

We further elucidated the potential relationships between CMRG scores and immune microenvironment characteristics in COAD using various immune infiltration assessment algorithms. ESTIMATE analysis indicated that in the low CMRG scoring subgroup, tumour purity scores were higher, while stroma and ESTIMATE scores were significantly lower (Figure 5A–D). Quantitative analysis of immune infiltrating cells based on the ssGSEA algorithm revealed that in the CMRG scoring subgroup B, the infiltration proportions of Immature dendritic cells, MDSC, Monocytes, Natural killer T cells, and Plasmacytoid dendritic cells were significantly increased (Figure 5E). Pearson correlation analysis demonstrated significant negative correlations between COX19 and all 23 types of immune infiltrating cells, whereas GPC1 showed significant positive correlations with most immune infiltrating cells (Figure 5F). By utilising the GDSC database, drug sensitivity analysis indicated that the low CMRG scoring subgroup had a higher IC50 for Salubrinal, implying lower sensitivity to the drug. In contrast, the high CMRG scoring subgroup might derive greater benefit from Salubrinal treatment. Conversely, in the low CMRG scoring subgroup, IC50 values for Erlotinib, Lapatinib, and Rapamycin were lower, indicating greater sensitivity to these drugs (Figure 5G). These results provide preliminary insights into the relationship between CMRG scoring subgroups and COAD immune microenvironment characteristics and predict potential clinical benefits of specific antitumor drugs for different CMRG scoring subgroups.

### 3.6 Somatic Mutation Characteristics and Immunotherapy Response Prediction of CMRG Scoring Subgroups in COAD

**FIGURE 6:**
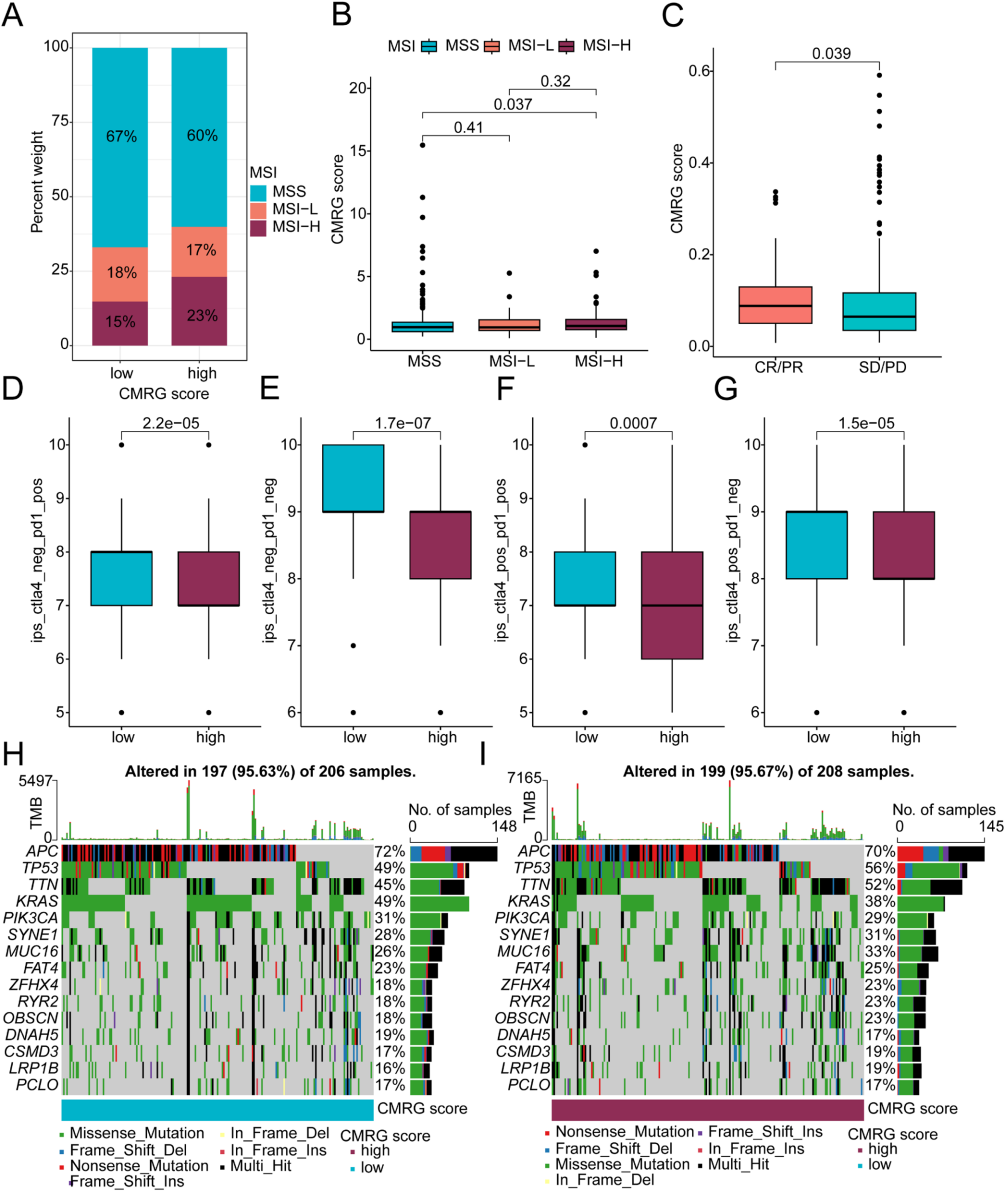
Somatic mutation landscape and immune therapy response evaluation. (A, B) Differential analysis of CMRG scores in MSI subgroups. (C) Assessment of PD‐L1 treatment response in CMRG scoring subgroups based on the IMvigor210 database. PR, Partial Response; PD, Progressive Disease; SD, Stable Disease; CR, Complete Response. (D‐G) IPS score predictions for CMRG scoring subgroups. (H, I) Somatic mutation landscape analysis of CMRG scoring subgroups.

We further explored the potential connections between CMRG scores and COAD microsatellite instability (MSI), somatic mutation characteristics, and immune therapy. In the CMRG scoring subgroups, we observed a higher proportion of MSS (microsatellite stable) subgroups in the low CMRG scoring subgroup and a lower proportion of MSI‐H (microsatellite instability‐high) subgroups. Additionally, MSI‐H subgroups had significantly higher CMRG scores compared to MSS subgroups (Figure 6A,B). In the CR/PR (complete response/partial response) subgroup, COAD samples with high CMRG scores had significantly higher scores compared to the SD/PD (stable disease/progressive disease) subgroup. This suggests that COAD samples with high CMRG scores may have better immune responses to PD‐L1 immune therapy (Figure 6C). Notably, IPS results revealed the response levels of COAD samples to PD1 and CTLA4 immune therapies. The results indicated that the low CMRG scoring subgroup might have better treatment responses to PD1 and CTLA4 therapies (Figure 6D–G). The somatic mutation landscape waterfall plots showed mutation frequencies in CMRG scoring subgroups. In the low CMRG scoring subgroup, high mutation frequencies were observed for APC (72%) and KRAS (49%). In contrast, the high CMRG scoring subgroup had higher mutation frequencies for TP53 (56%), TTN (52%), SYNE1 (31%), and MUC16 (33%) (Figure 6H,I). Based on these results, we have preliminarily clarified the potential associations between CMRG scoring subgroups and COAD immunotherapy, providing new strategies and insights for immune therapy in different COAD risk subgroups.

### 3.7 Single‐Cell Sequencing Analysis Reveals the Distribution Characteristics of CMRG Signatures

**FIGURE 7:**
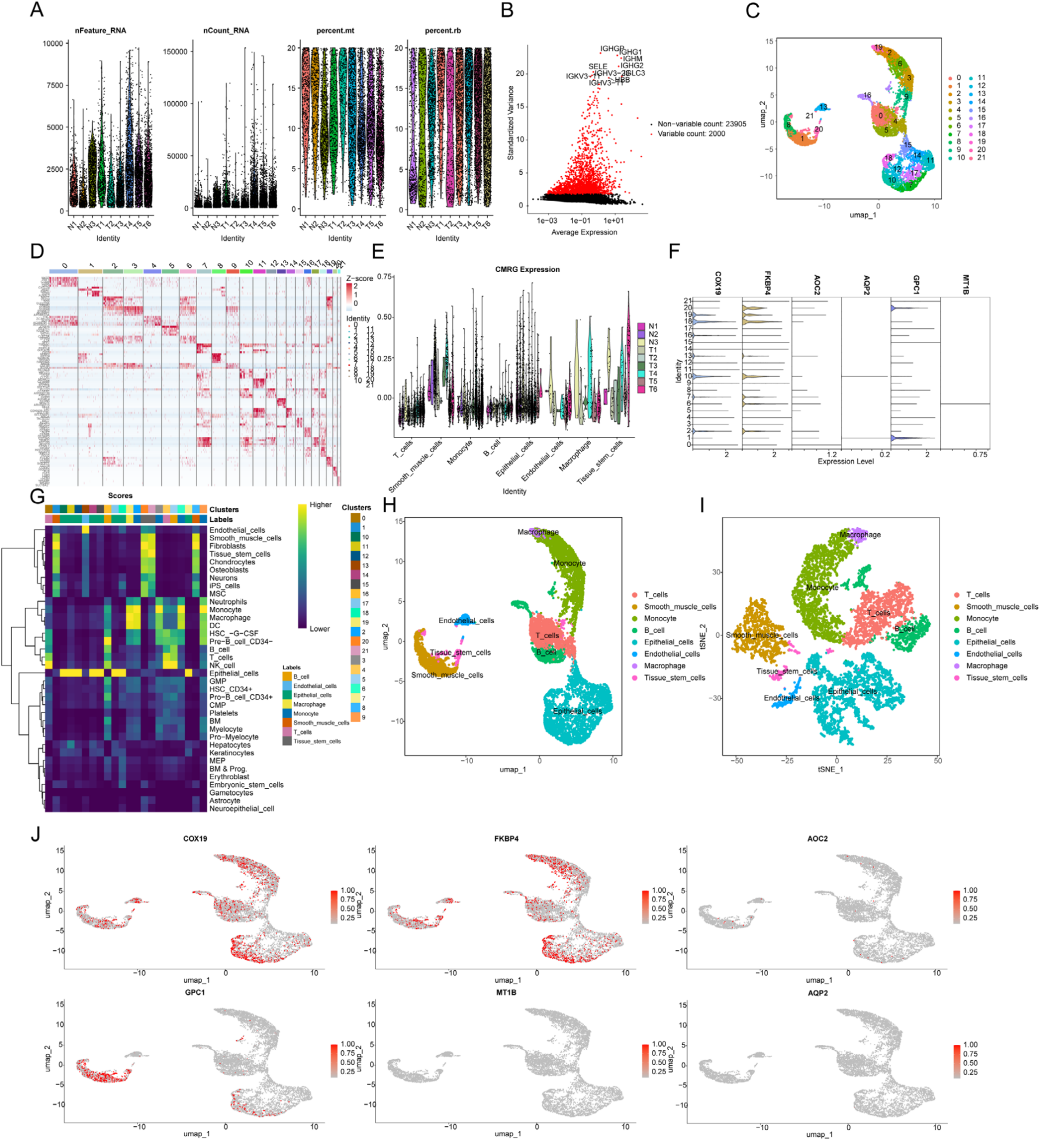
Single‐cell sequencing analysis of CMRG prognostic signatures. (A) Normalisation and quality control of the single‐cell dataset GSE231559. (B) Identification of highly variable genes. (C) Cell subtype identification based on UMAP dimensionality reduction analysis. (D) Heatmap of marker gene expression across 21 cell subtypes. (E) Expression of CMRG signatures in 8 cell subtypes. (F) Expression levels of CMRG prognostic signatures across 21 cell subtypes. (G) Cell subtype annotation and scoring based on SingleR. (H, I) UMAP and t‐SNE dimensionality reduction analysis revealing distribution characteristics of 8 cell subtypes. (J) UMAP plot illustrating the localization and expression features of 6 CMRG prognostic signatures within cell subtypes.

To spatially resolve the expression patterns of prognostic CMRG, we performed single‐cell sequencing analysis in COAD tissues. Using the GSE231559 dataset, we extracted 3 adjacent normal samples and 6 COAD tissue samples for single‐cell sequencing analysis. After normalisation and quality control of the data from the 9 samples, we obtained 2000 highly variable genes and 23,905 other genes (Figure 7A,B). We conducted dimensionality reduction analysis using UMAP, accurately identifying 21 cellular subpopulations in COAD (Figure 7C). A heatmap illustrating the expression of marker genes across the 21 cellular subpopulations is shown (Figure 7D). As shown in Figure 7E, a violin plot shows the expression distribution of CMRG signatures across 78 cell types in adjacent normal and COAD tissue samples. Additionally, among the 21 cellular subpopulations, COX19, FKBP4, and GPC1 exhibited relatively high expression levels (Figure 7F). We annotated these 21 cellular subpopulations using the SingleR algorithm, resulting in 8 cell types: T cells, smooth muscle cells, monocyte, B cells, epithelial cells, endothelial cells, macrophage, and tissue stem cells (Figure 7G). UMAP and tSNE dimensionality reduction analyses further elucidated the distribution of the 8 cell types (Figure 7H,I). Furthermore, UMAP analysis assessed the distribution of 6 CMRG prognostic signatures across the 8 cell types, revealing that COX19 and FKBP4 were highly expressed across all 8 cell types. GPC1 showed high expression levels specifically in Smooth muscle cells and Epithelial cells (Figure 7J). These findings provide insights into the spatial and cellular distribution of CMRG signatures within COAD tissue, shedding light on their roles in different cell types and their potential as therapeutic targets.

### 3.8 COX19 Knockdown Significantly Inhibits Proliferation and Invasion of COAD

**FIGURE 8:**
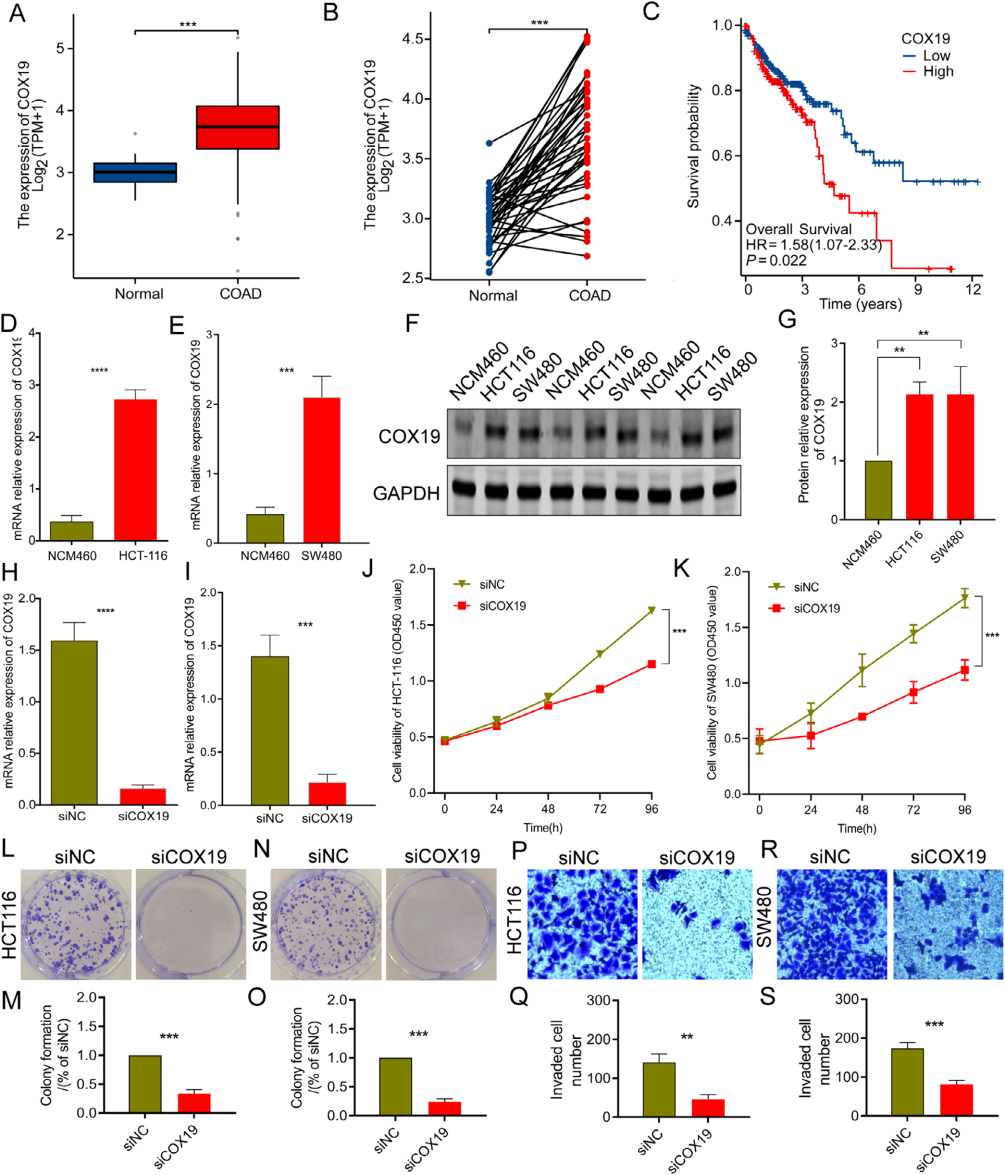
Interference with COX19 expression significantly inhibits invasion and proliferation of COAD. (A, B) Differential expression and paired analysis of COX19 between normal and tumour samples in the TCGA database. (C) Survival curve analysis of COX19 expression subgroups. (D, E) mRNA expression analysis of COX19 in normal cell line (NCM460) and COAD cell lines (HCT‐116, SW480). (F, G) Quantitative analysis of COX19 protein expression in normal cell line (NCM460) and COAD cell lines (HCT‐116, SW480). (H, I) Evaluation of COX19 interference efficiency in HCT‐116 and SW480 cell lines. (J, K) CCK8 assay in HCT‐116 and SW480 cell lines. (L–S) Colony formation assay and Transwell experiment analysis of HCT‐116 and SW480 cell lines (*n* = 3) (×100).

Single‐cell sequencing analysis revealed that COX19 is highly expressed across various cell subtypes in COAD. Given that COX19 has the highest risk coefficient among the CMRG prognostic signatures based on multivariate Cox analysis, it suggests that COX19 could be a key regulator in COAD. Therefore, we further explored the potential relationship between COX19 and COAD. Using the TCGA database, we found that COX19 is significantly overexpressed in COAD samples compared to normal samples, and paired sample analysis also indicated that COX19 expression is markedly higher in COAD tissues than in adjacent normal tissues (Figure 8A,B). Survival curve analysis revealed that COX19 high‐expression subgroup had significantly worse survival outcomes compared to the low‐expression subgroup in COAD (Figure 8C, HR = 1.58, *p* = 0.022), suggesting that COX19 is associated with poor prognosis in COAD. In vitro experiments showed that COX19 mRNA expression was significantly higher in the HCT‐116 and SW480 cell lines compared to the NCM460 cell line, as indicated by qPCR analysis (Figure 8D,E). Western blot results confirmed that COX19 protein levels were also significantly elevated in HCT‐116 and SW480 cells (Figure 8F,G). To further explore COX19's role in COAD progression, we silenced its expression in the HCT‐116 and SW480 cell lines using siRNA. qRT‐PCR analysis showed a significant reduction in COX19 expression in HCT‐116 and SW480 cells compared to the negative control group (Figure 8H,I). CCK8 assay assessed the proliferation of HCT‐116 and SW480 cells at 24‐, 48‐, 72‐, and 96‐h post‐transfection. The results showed a significant decrease in cell proliferation at 72 and 96 h after COX19 knockdown (Figure 8J,K). Additionally, colony formation and invasion assays indicated a marked reduction in the colony‐forming ability and invasive capacity of COX19‐interfered COAD cells (Figure 8L–S). In summary, our in vitro data suggest that inhibiting COX19 expression impacts COAD cell proliferation and invasion, which may influence disease progression.

## 4 Discussion

By screening prognostically relevant CMRG, our results highlight the potential role of copper metabolism in COAD pathogenesis. Elevated serum copper levels were strongly associated with colorectal cancer malignancy, including COAD [28]. KRAS mutations, which may be present in COAD patients, promote a large influx of copper into the cell, thereby supporting the proliferation of malignant cells [29]. In addition, multiple cytokines have been reported to promote intestinal tumorigenesis through regulation of copper uptake. For example, IL‐17 enhances the activation of the NF‐κB cancer‐promoting pathway by inducing STEap4‐dependent cytoplasmic copper uptake [30]. In addition to the effect on cancer cell proliferation, the effect of copper metabolism on COAD tumour metastasis has also been emphasised. Copper and copper‐binding proteins facilitate COAD metastasis by activating transcription factors such as ZEB1, ZEB2, and Snail during epithelial‐mesenchymal transition (EMT) [31, 32]. Therefore, our risk stratification of COAD patients based on copper metabolism has a theoretical basis and potential application value.

While copper is essential for cell survival and proliferation and serves as a potential therapeutic target in cancer, excess copper can be toxic by generating harmful ROS [33]. A recently discovered form of copper‐dependent programmed cell death, known as cuproptosis, occurs when excess intracellular copper binds to fatty acylated proteins in the mitochondrial tricarboxylic acid (TCA) cycle, leading to protein toxicity stress and ultimately cell death [34, 35, 36]. There is evidence that a key protein of cuproptosis is associated with COAD prognosis [37]. Therefore, copper ionophores such as disulfiram and elesclomol have strong theoretical support for inducing cuproptosis in tumours, including COAD [38, 39, 40, 41]. In addition, both disulfiram and elesclomol have been reported to enhance tumour immune processes by influencing the immune microenvironment or binding to PD‐L1 [42, 43]. The synergistic effect of cuproptosis‐related nanoparticles with immunotherapy was further demonstrated to enhance the efficacy of cancer immunotherapy [44]. Therefore, targeting copper metabolic processes has great potential for anti‐tumour therapy.

Our results suggest the important role of COX19 in the development of COAD. Cytochrome c oxidase 19 (COX19) exists in the cytoplasm and membrane space of mitochondria, and its role is to transport Cu within the mitochondria [45, 46]. COX19, a target gene of MACC1, regulates mitochondrial activity and drives tumour progression in colorectal cancer [47]. In addition, in mitochondria, the dynamic REDOX‐regulated interaction between Cox19 and the copper‐binding protein Cox11 promotes the production of cytochrome c oxidase (COX) [48]. COX can produce reactive oxygen species (ROS) in mitochondria under stress [49]. It has been reported that in colon cancer patients, increased COX content in mitochondria can reduce the sensitivity of cancer cells to apoptotic stimulation and support cell growth/survival [50]. Drugs targeting COX have potential clinical antitumor properties [51]. In the results of single cell sequencing, we also observed the increased expression of COX19 in immune cells. However, current literature lacks direct evidence linking COX19 to immune cell regulation. Given the crucial role of ROS in the tumour microenvironment and its potential impact on tumour immunotherapy, further research on the relationship between COX19 and immune cells is warranted. Such studies will enhance our understanding of tumour‐related immunotherapy [52, 53].

Formation of neoplastic immunosuppressive microenvironment and immune escape play an important role in the pathogenesis of colorectal cancer [54]. In addition to targeting cancer cells by directly releasing cytotoxic molecules including perforin and granase [55], the active role of NK cells in cancer immune surveillance has also been demonstrated [56]. However, in colorectal cancer, including COAD, the role of NK cells remains unclear as they can exert both anti‐tumour and pro‐tumour effects [57]. NK cells in colorectal tumours produce IFN‐γ and increase IFN‐γ levels in the tumour [57]. IFN‐γ also promotes tumour escape by up‐regulating the expression of PD‐L1 on tumour cells [58, 59, 60]. Therefore, the role of NK cells in the development of COAD remains to be fully characterised. Our immunoinfiltration results showed that higher levels of NK cell infiltration in the tumour microenvironment were associated with poorer prognosis, which also suggested a possible tumour‐promoting role of NK cells. Further analysis of the role of NK cells in different stages of COAD development in the future will help to better understand the function of NK cells.

In summary, we demonstrated the feasibility and reliability of risk stratification of COAD patients based on copper metabolism and preliminary screening of potential intervention targets. However, the public database is the main data source, so the results in this paper are mostly correlation analysis rather than causation analysis. Although the important value of public databases in risk stratification of clinical oncology patients has been proposed, further large‐scale cohort validation is still necessary [61, 62, 63]. This study was validated solely based on TCGA and GEO data. Future research could expand to external validation using ICGC or other high‐quality cancer databases to enhance the model's applicability. Moreover, our understanding of the underlying molecular mechanisms remains limited. Subsequent studies may integrate more colorectal cancer cohorts or even conduct cross‐cancer validation to assess the model's generalisability, thereby strengthening its potential for clinical translation.

## Author Contributions

**Xi Sun:** investigation (equal), writing – original draft (equal). **Jingfei Tong:** writing – review and editing (equal). **Xiaojie Fang:** formal analysis (equal), methodology (equal). **Miaojiong Lu:** software (equal). **Chunhui Rao:** validation (equal), writing – review and editing (equal). **Yanyan Li:** validation (equal), visualization (equal).

## Ethics Statement

The authors have nothing to report.

## Consent

The authors have nothing to report.

## Conflicts of Interest

The authors declare no conflicts of interest.

## Supporting information


Appendix S1.

## Data Availability

The data that support the findings of this study are available from the corresponding author upon reasonable request.
