# Supplementary material for: Comprehensive Multi‐Omics Analysis of Copper Metabolism Related Molecular Subtypes and Prognostic Risk Stratification in Colon Adenocarcinoma
Source: J Cell Mol Med. 2025 May 20;29(10):e70591. doi: 10.1111/jcmm.70591 (PMC12089994; doi:10.1111/jcmm.70591)
Supplement: Supplementary file 1 — Appendix S1. [file JCMM-29-e70591-s001.docx]

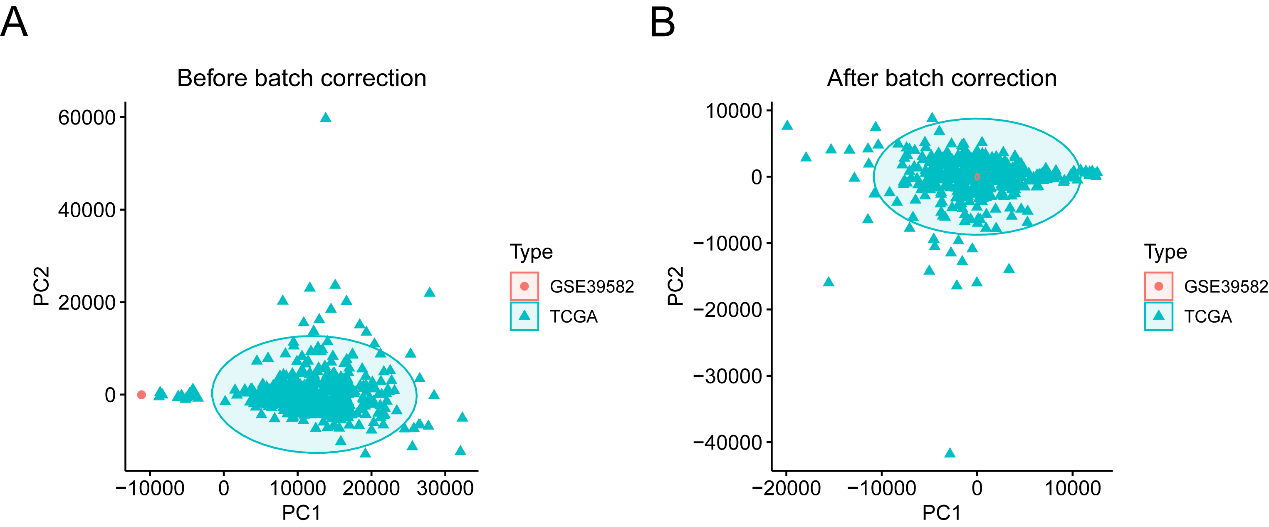


Supplementary Figure 1. Removal of batch effects in transcriptomic data from TCGA and GEO cohorts. (A) Principal component analysis (PCA) plot before batch effect correction. (B) PCA plot after batch effect correction.


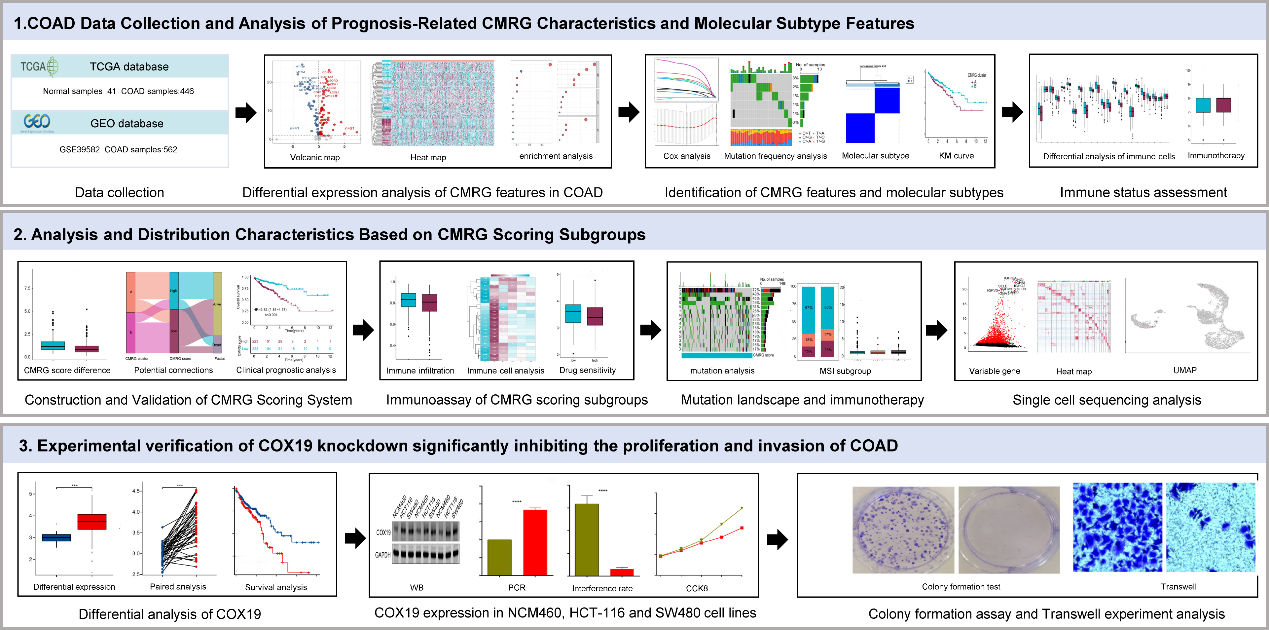


Supplementary Figure 2. The workflow diagram of this study.


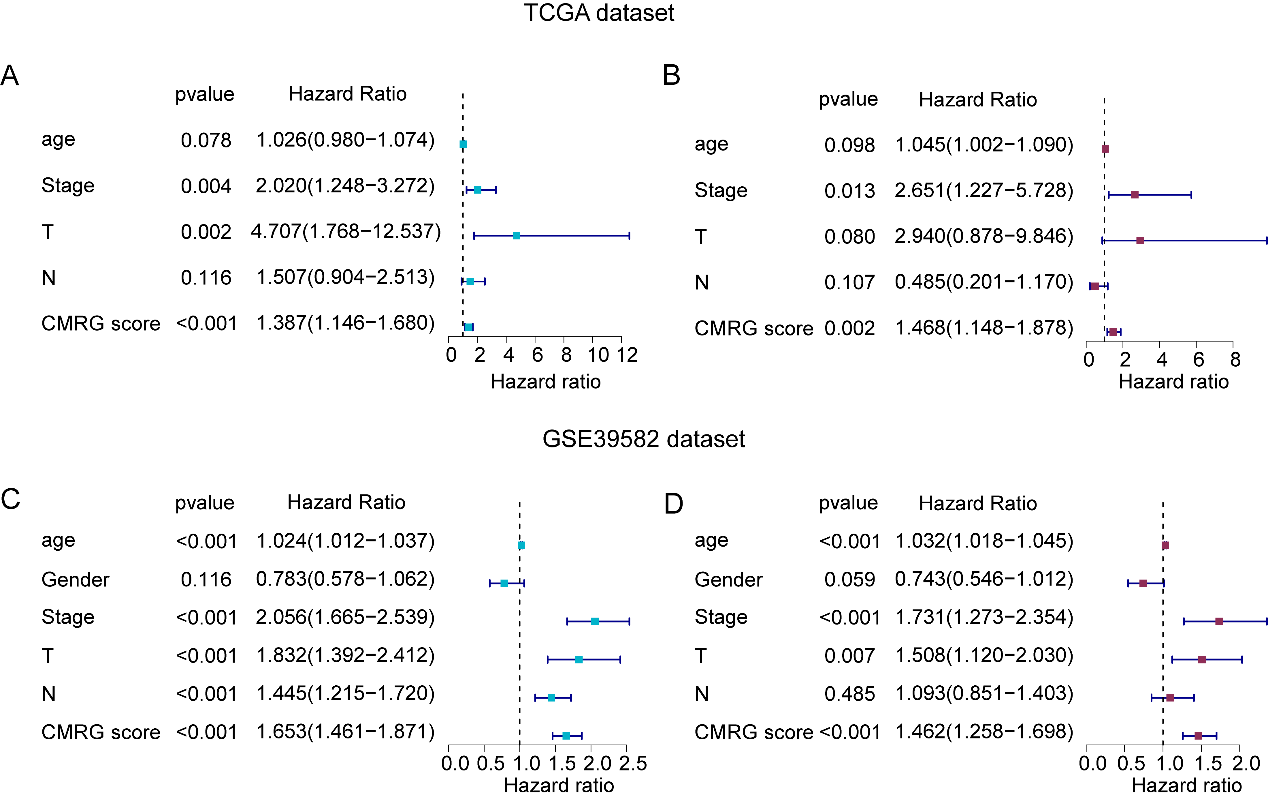


Supplementary Figure 3. Independent prognostic analysis of clinical pathological variables and CMRG scores index. (A, B) Univariate and multivariate Cox analysis of clinical pathological variables and CMRG score in the TCGA dataset. (C, D) Univariate and multivariate Cox analysis of clinical pathological variables and CMRG score in the GSE39582 dataset.

Supplementary Table 1. The gene list of copper metabolism-related genes.

| Gene symbol |
| --- |
| ABCB6 |
| ANKRD9 |
| SLC31A1 |
| SLC31A2 |
| PRND |
| CCDC22 |
| APP |
| ARF1 |
| MT2A |
| ATOX1 |
| ATP7A |
| ATP7B |
| PRNP |
| SCO1 |
| COX19 |
| SCO2 |
| CYP1A1 |
| DAXX |
| BACE1 |
| AOC1 |
| MT1DP |
| HSF1 |
| AQP1 |
| AQP2 |
| MT1A |
| MT1B |
| MT1E |
| MT1F |
| MT1G |
| MT1H |
| MT1M |
| MT1X |
| MT3 |
| NFE2L2 |
| MT1HL1 |
| SNCA |
| MAP1LC3A |
| MT4 |
| BECN1 |
| COMMD1 |
| XIAP |
| CUTC |
| STEAP2 |
| STEAP3 |
| STEAP4 |
| SLC11A2 |
| COX17 |
| CP |
| FKBP4 |
| HEPHL1 |
| MMGT1 |
| HEPH |
| PARK7 |
| AANAT |
| IL1A |
| LCAT |
| LOXL2 |
| MT-CO1 |
| PAM |
| ATP5F1D |
| SOD1 |
| SOD3 |
| SORD |
| TFRC |
| CDK1 |
| MOXD2P |
| MTCO2P12 |
| COX11 |
| LACC1 |
| DBH |
| DCT |
| ALB |
| F5 |
| F8 |
| OR5AR1 |
| ADNP |
| ATP13A2 |
| MOXD1 |
| GPC1 |
| ANG |
| SUMF1 |
| AOC2 |
| SNAI3 |
| APOA4 |
| COA6 |
| LOX |
| LOXL1 |
| MT-CO2 |
| ACR |
| P2RX4 |
| CUTA |
| HAMP |
| S100A5 |
| S100A12 |
| S100A13 |
| SNCB |
| SNCG |
| TP53 |
| TYR |
| LOXL4 |
| LOXL3 |
| AOC3 |
| RNF7 |
| CCS |
| AP1S1 |
| AP1B1 |
| TMPRSS6 |
| SPATA5 |
| COG2 |
| ATP6V0A2 |
| ATP6AP1 |
| ADAM10 |
| AKT1 |
| MTF2 |
| FOXO1 |
| FOXO3 |
| STEAP1 |
| GSK3B |
| APC |
| JUN |
| MAPT |
| MDM2 |
| MT1JP |
| MT1L |
| MTF1 |
| PIK3CA |
| XAF1 |
| PTEN |
| CCND1 |
| SP1 |
| ADAM17 |
| CASP3 |
| ADAM9 |
